# Aripiprazole in the Treatment of Irritability in Children and Adolescents with Autism Spectrum Disorder in Japan: A Randomized, Double-blind, Placebo-controlled Study

**DOI:** 10.1007/s10578-016-0704-x

**Published:** 2016-12-21

**Authors:** Hironobu Ichikawa, Katsunaka Mikami, Takashi Okada, Yushiro Yamashita, Yuko Ishizaki, Akemi Tomoda, Hiroaki Ono, Chiharu Usuki, Yoshihiro Tadori

**Affiliations:** 1 Tokyo Metropolitan Children’s Medical Center, Fuchu, Tokyo Japan; 2 Department of Psychiatry, Tokai University School of Medicine, Isehara, Kanagawa Japan; 3 Department of Child and Adolescent Psychiatry, Nagoya University Graduate School of Medicine, Nagoya, Aichi Japan; 4 Department of Pediatrics and Child Health, Kurume University School of Medicine, Kurume, Fukuoka Japan; 5 Department of Pediatrics, Kansai Medical University Medical Center, Moriguchi, Osaka Japan; 6 Research Center for Child Mental Development, University of Fukui, Fukui, Japan; 7 Department of Clinical Research and Development, Otsuka Pharmaceutical Co., Ltd., Minato-ku, Tokyo, Japan; 8 Department of Medical Affairs, Otsuka Pharmaceutical Co., Ltd., Minato-ku, Tokyo, Japan

**Keywords:** Aripiprazole, Autism spectrum disorder, Irritability, Children and adolescent

## Abstract

We evaluated the efficacy and safety of aripiprazole in the treatment of irritability in children and adolescents (6–17 years) with autism spectrum disorder (ASD) in a randomized, double-blind, placebo-controlled 8-week study in Japan. Patients received flexibly dosed aripiprazole (1–15 mg/day) or placebo. Ninety-two patients were randomized to placebo (n = 45) or aripiprazole (n = 47). Aripiprazole produced a significant improvement in the mean parent/caregiver-rated Aberrant Behavior Checklist Japanese Version irritability subscale score relative to placebo from week 3 through week 8. Administration of aripiprazole provided significantly greater improvement in the mean clinician-rated Clinical Global Impression-Improvement scores than placebo from week 2 through week 8. All patients randomized to aripiprazole completed the study, and no serious adverse events were reported. Three patients in placebo group discontinued. Aripiprazole was effective and generally safe and well-tolerated in the treatment of irritability associated with ASD in Japanese children and adolescents.

## Introduction

Before 2013, autistic spectrum disorders (ASD) represented pervasive developmental disorders of variable severity, defined as autistic disorder, Asperger’s disorder and pervasive developmental disorder—not otherwise specified (PDD-NOS) in the American Psychiatric Association’s Diagnostic and Statistical Manual of Mental Disorders (DSM), Fourth Edition, Text Revision (DSM-IV-TR) [1]. The three characteristic manifestations of ASD are (1) impaired social interaction, (2) impaired communication and (3) restricted repetitive and stereotyped patterns of behavior, activities, or interests. Diagnostic criteria for ASD changed significantly with release of the fifth edition of the DSM (DSM-5) in 2013 [2]. Autistic disorder, Asperger’s disorder and PDD-NOS were collapsed into a single diagnosis of ASD—a single diagnosis with considerable diagnostic variability. The social and communication domains of ASD were combined, leaving two key symptom domains: (1) social communication and (2) restricted and repetitive behaviors. Understandably, these symptoms can have a substantial impact on affected individuals and their families. This impact can be further increased by the presence of associated behaviors such as irritability, which may manifest as tantrums, aggressiveness, self-injurious behavior, and sudden mood changes, all of which can have a significant impact on education and social development [3].

Although there are no approved pharmacologic treatments that target the core deficits of ASD, associated comorbid symptoms such as irritability may be ameliorated by a combination of behavioral and pharmacologic approaches, including the use of atypical antipsychotics [4]. Risperidone and aripiprazole are approved by the US Food and Drug Administration for the treatment of pediatric patients with irritability associated with ASD.

Aripiprazole is an atypical antipsychotic with a partial agonism at dopamine D_2_ receptors and serotonin 5-HT_1A_ receptors and an antagonism at 5-HT_2A_ receptors [5, 6]. Aripiprazole may have a more favorable side-effect profile than other antipsychotics in child and adolescent patients with mental health disorder [7], because of its unique mechanism of action. Aripiprazole was shown to be efficacious while generally safe and well tolerated for the treatment of children and adolescents (ages 6–17 years) with irritability associated with ASD in two 8-week, double-blind, randomized, placebo-controlled studies and in a 52-week open-label flexible-dose study in the United States [8–12].

To our knowledge, no controlled studies have tested aripiprazole for use in ASD in Asia including Japan. The results of the US study conducted predominantly in Caucasian patients may not be generalizable to Japanese children with ASD. As genetic/physiologic variation can result in differences in metabolism of antipsychotic medications, response to treatment and adverse-event risk. Behaviors associated with autistic traits were reported to a greater extent in the Eastern cultures than the Western culture [13]. It is important to study new treatment options in specific populations and/or different cultures and environments from the viewpoint of the possible existence of ethnic differences.

Before 2016, in Japan, the typical antipsychotic pimozide was the only approved medication for the treatment of pediatric patients with irritability associated with ASD. A phase 3 clinical study of aripiprazole in Japan was needed to obtain approval for this indication from Pharmaceuticals and Medical Devices Agency. We conducted a multicenter, randomized, double-blind, placebo-controlled, 8-week phase 3 study to confirm the efficacy and safety profiles of flexibly dosed aripiprazole (1–15 mg/day) in children and adolescents with irritability associated with ASD in Japan.

## Methods

This multicenter, randomized, double-blind, placebo-controlled phase 3 study was conducted at 50 sites in Japan between June 2012 and June 2015 in accordance with ethical principles originating from the Declaration of Helsinki and in compliance with International Conference on Harmonization Good Clinical Practice guideline. The institutional review board at each site approved the protocol. All parents/guardians provided written informed consent to participate in the study, and patients provided written informed assent when possible. The study was registered at ClinicalTrials.gov (identifier: NCT01617447).

### Subjects

Eligible patients were children and adolescents aged 6–17 years with a diagnosis of autistic disorder (not ASD) defined by the DSM-IV-TR criteria, and with behavioral problems such as irritability, agitation, self-injurious behavior, or a combination of these symptoms, and with a Clinical Global Impression-Severity of Illness scale (CGI-S) score of ≥4 and an Aberrant Behavior Checklist Japanese Version (ABC-J) score [14] of ≥18 at screening and baseline. The pervasive developmental disorders autism society Japan rating scale (PARS) [15] was used as an assessment of autistic disorder, not ASD. The validity and reliability of ABC-J are equivalent to the original ABC [16] and it is useful for assessing behavior problems in Japanese patients with intellectual disability [14].

Patients who had complications or histories of schizophrenia, other psychosis, and mood disorders including bipolar disorder and major depression according to the DSM-IV-TR criteria were excluded. Patients were diagnosed by the investigator. Other exclusion criteria included a diagnosis of Rett disorder, childhood disintegrative disorder, Asperger’s disorder, or pervasive development disorder not otherwise specified according to the DSM-IV-TR, or a diagnosis of fragile X syndrome. Other exclusion criteria included treatment resistance to antipsychotic medication, significant risk of committing suicide, or a profound intellectual disability. Patients who had previously used aripiprazole, who received any investigational drug within 30 days before providing informed consent, or who received any concomitant drug or therapy specified as prohibited in the study protocol from the designated time point onward were also excluded.

Presence or absence of intellectual disability was diagnosed based on the DSM-IV-TR. If intellectual disability was diagnosed, the severity was classified into the three categories: mild intellectual disability [intelligence quotient (IQ) level: 50–55 to approximately 70], moderate intellectual disability (IQ level: 35–40 to 50–55), severe intellectual disability (IQ level: 20–25 to 35–40). IQ was measured by one of the standardized methods.

#### Study Design

This study consisted of a 4-week screening phase and an 8-week treatment phase. Patients attended a screening visit and a baseline visit. Subsequently, study visits were designed at weeks 1, 2, 3, 4, 5, 6, and 8 or at the time of early discontinuation. The screening examination was conducted during the screening phase to confirm the eligibility of each patient. Patients who met inclusion criteria at baseline were randomized to receive aripiprazole or placebo (1:1). Clinicians were required to input information regarding eligible patients on the Interactive Web Response System (IWRS), and then the registration center assigned a trial drug code to each patient. The investigators and subjects were blinded to the trial drug randomization code. Aripiprazole was initiated at 1 mg/day, with a target dosage of 1, 3, 6, 9, 12, or 15 mg/day. The current dose was up-titrated in one week intervals to the next higher dose according to the patient’s tolerability when the Clinical Global Impressions-Improvement (CGI-I) score was assessed as ≥3. The dose was fixed at week 6 and maintained until week 8. The dose could be down-titrated any time at the clinician’s discretion according to tolerability at the current dose. Subsequent up-titration was permitted, but patients who experienced a second down-titration were excluded from the trial. A final examination was conducted at week 8, at the time of treatment completion. If patients prematurely discontinued the trial for any reason, the same examination was conducted at the time of discontinuation.

Concomitant medications such as antipsychotics, psychostimulants, mood stabilizers, antidepressants, antiepileptics, hypnotics and anxiolytics were prohibited during the trial. Patients were allowed to receive ultrashort-acting non-benzodiazepine hypnotic agents (i.e. zopiclone, zolpidem and triclofos sodium) as well as the melatonin receptor agonist ramelteon at the clinician’s discretion. Anticholinergic antiparkinson drugs (i.e. biperiden and trihexyphenidyl) were permitted for the treatment of extrapyramidal symptoms (EPS).

#### Assessments

Efficacy and safety assessments were performed at each study visit, and, when applicable, at the time of early termination. The primary endpoint was the mean change in the caregiver-rated ABC-J irritability subscale score [14] from baseline to week 8. The ABC-J irritability subscale is consist of 15 items that include such as “injures self”, “physical violence to self”, “aggressive to other children and adults”, “irritable,” “temper outbursts”, “depressed mood”, “mood change”, and “yells” or “screams” etc. Individual items are rated from 0 (never a problem) to 3 (severe problem). The secondary endpoints included the clinician-rated mean CGI-I score, the response rate (≥25% reduction from baseline in the ABC-J irritability subscale score and a CGI-I score of 1 or 2), the mean change in the other ABC-J subscale scores (lethargy/social withdrawal (16 items), stereotypy (7 items), hyperactivity (16 items), and inappropriate speech (4 items) from 0 to 3) and the response rate of ABC-J score (ABC-J response rate: ≥50% reduction from baseline in at least 2 subscales and <10% increase from baseline in the remaining subscales) from baseline to week 8. The CGI-I scale quantifies the clinician’s impression of the patient’s improvement or worsening relative to baseline, scoring from one (very much improved) to seven (very much worse).

Additional secondary endpoints included the mean changes in the CGI-S score, the Children’s Yale-Brown Obsessive–Compulsive Scale (CY-BOCS, compulsion scale only) score [17], and the Children’s Global Assessment Scale (CGAS) score [18] from baseline to week 8. The CGI-S scale quantifies the clinician’s impression of the patient’s current illness severity on a scale from one (normal, not at all ill) to seven (among the most extremely ill patients). The CY-BOCS is a semi-structured measure of obsessive–compulsive symptom severity in children and adolescents. Investigators selected compulsive behaviors of patients and scored the severities of these behaviors with time during the behavior, interference due to the behavior, distress associated with the behavior, resistance against compulsions and degree of control over compulsive thoughts, from 0 (none) to 4 (extreme). CGAS is 100-point rating scale measuring psychological, social and school functioning for children aged 6–17. Increase in scores of CGAS reflects improvement in symptoms. The CY-BOCS and CGAS scores were measured at baseline and at weeks 4 and 8.

Safety assessment measures, which included adverse event (AEs) data, vital signs, body weight, and Columbia Suicide Severity Rating Scale (C-SSRS), were collected at each visit. In addition, the presence and the severity of EPS were assessed at each visit by the investigator using the Drug Induced Extra-Pyramidal Symptoms Scale (DIEPSS), the Abnormal Involuntary Movement Scale (AIMS) and the Barnes Akathisia Rating Scale (BAS). A12-lead ECG and laboratory tests were performed at baseline and at the end of treatment.

#### Statistical Analysis

The randomized sample included all subjects who were randomized to double-blind treatment. The safety sample included all randomized subjects who took at least one dose of study medication during the double-blind treatment phase, and the efficacy sample included all subjects in the safety sample who had at least one post-randomization efficacy evaluation and corresponding baseline value. For continuous measurement, change scores were evaluated by analysis of covariance (ANCOVA). The ANCOVA models for last observation carried forward (LOCF) data sets included the baseline measure as a covariate and baseline body weight (≥40 kg or <40 kg), and treatment as a priori main effects. *P* values that were generated from the ANOVA model tested the least square means treatment differences (TDs). For the treatment response rate and the ABC-J response rate, the statistical comparison between groups was conducted using Pearson’s chi square analysis. The proportion of the response rate of aripiprazole to that of placebo and the two-sided 95% confidence interval (CI) were calculated.

All statistical analyses were performed using the SAS software (version 9.2; SAS Institute Inc.). For the evaluation of efficacy, data values were fundamentally expressed as mean (standard error: SE), and statistical analysis results were represented as P value and 95% CI. Statistical differences were considered significant when P < 0.05 (two-sided). Evaluations of prolactin concentrations, weight and body mass index (BMI) changes from baseline to week 8 were performed using observed case (OC) data set.

Considering alpha = 0.05, beta = 0.15, and seven score difference between the aripiprazole and the placebo groups on the mean changes of irritability subscale from ABC-J, the required sample size was estimated as a minimum of 32 patients in each group.

## Results

### Subject Disposition and Demographics

**Fig. 1:**
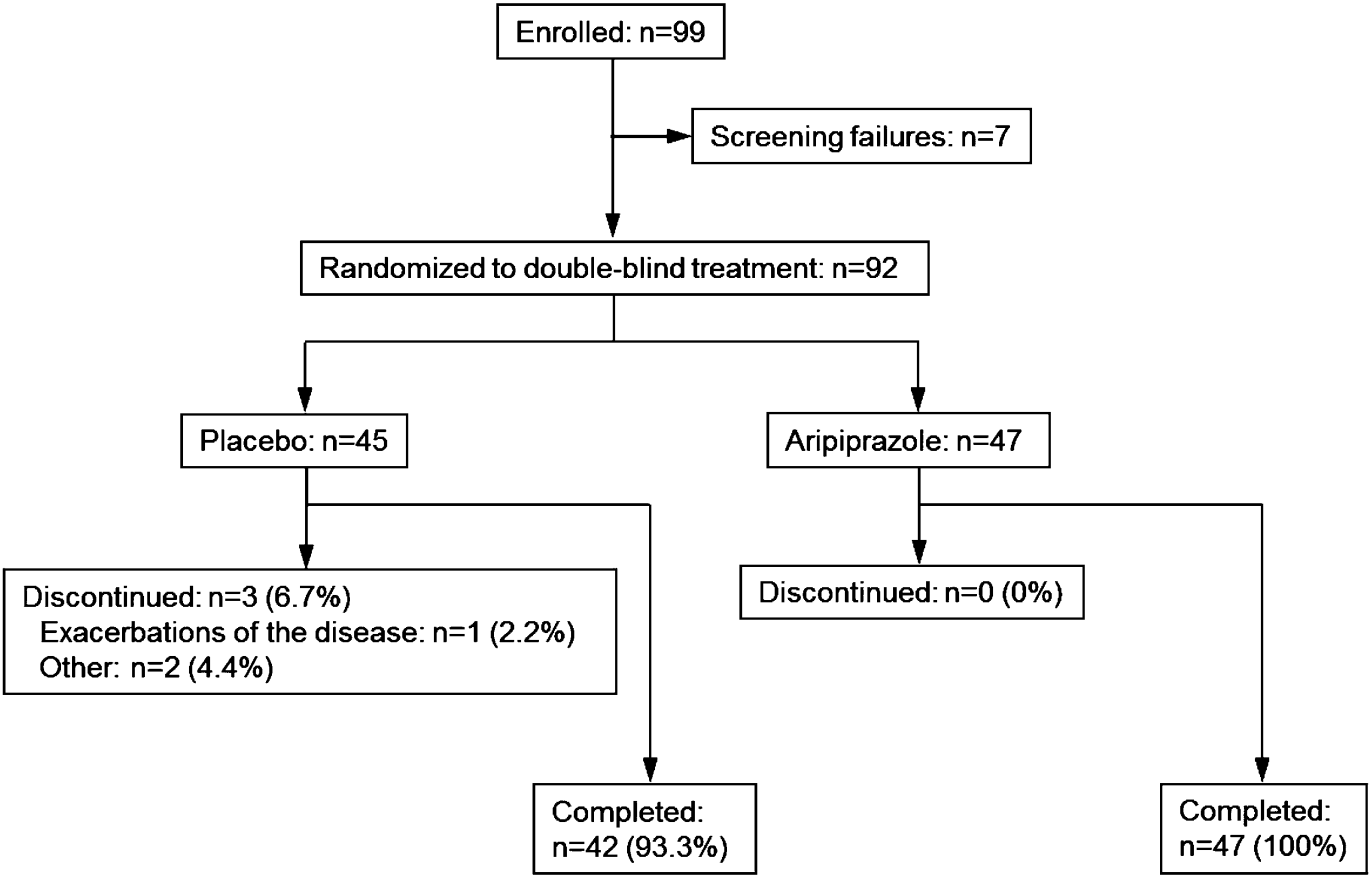
Subject disposition

In total, 99 patients were enrolled and 92 patients were randomly assigned to receive placebo (n = 45) or aripiprazole (n = 47). Forty-two (93.3%) patients in the placebo group and 47 (100%) patients in the aripiprazole group completed the trial; subject disposition is shown in Fig. 1. All randomized patients (n = 92) were included the efficacy and safety sample.

The demographic and clinical characteristics at baseline are shown in Table 1. The mean age of the randomized patients was 10.1 years old, and the majority of patients were males and younger than 13 years. Fifty-eight (63.0%) patients with intellectual disability were included in the trial.

**Table 1:** Baseline demographics and clinical characteristics

	Placebo (n = 45)	Aripiprazole (n = 47)	Total (n = 92)
Gender			
Male	36 (80.0)	39 (83.0)	75 (81.5)
Female	9 (20.0)	8 (17.0)	17 (18.5)
Age, years^a^	9.9 (3.1)	10.3 (3.3)	10.1 (3.2)
6–12 years	36 (80.0)	33 (70.2)	69 (75.0)
13–17 years	9 (20.0)	14 (29.8)	23 (25.0)
Height, cm^a^	137.6 (18.1)	140.7 (19.0)	139.2 (18.5)
Weight, kg^a^	36.4 (15.5)	39.1 (17.6)	37.8 (16.5)
<40 kg	31 (68.9)	26 (55.3)	57 (62.0)
≥40 kg	14 (31.1)	21 (44.7)	35 (38.0)
Body mass index, kg/m^2a^	18.4 (3.8)	18.7 (4.2)	18.6 (4.0)
Intellectual disability^b^	29 (64.4)	29 (61.7)	58 (63.0)
Intelligence quotient (IQ)^c^			
Mild	16 (35.6)	16 (34.0)	32 (34.8)
Moderate	7 (15.6)	7 (14.9)	14 (15.2)
Severe	6 (13.3)	6 (12.8)	12 (13.0)
ABC-J irritability subscale score^a^	26.8 (6.5)	27.1 (7.2)	27.0 (6.9)
CGI-S^a^	5.0 (0.8)	4.9 (0.7)	4.9 (0.8)

Data are expressed as number (%)

^a^Mean (standard deviation)

^b^Intellectual disability was diagnosed based on DSM-IV-TR

^c^Mild (IQ level: 50–55 to approximately 70), moderate (IQ level: 35–40 to 50–55), severe (IQ level: 20–25 to 35–40)

### Study Medication

**Fig. 2:**
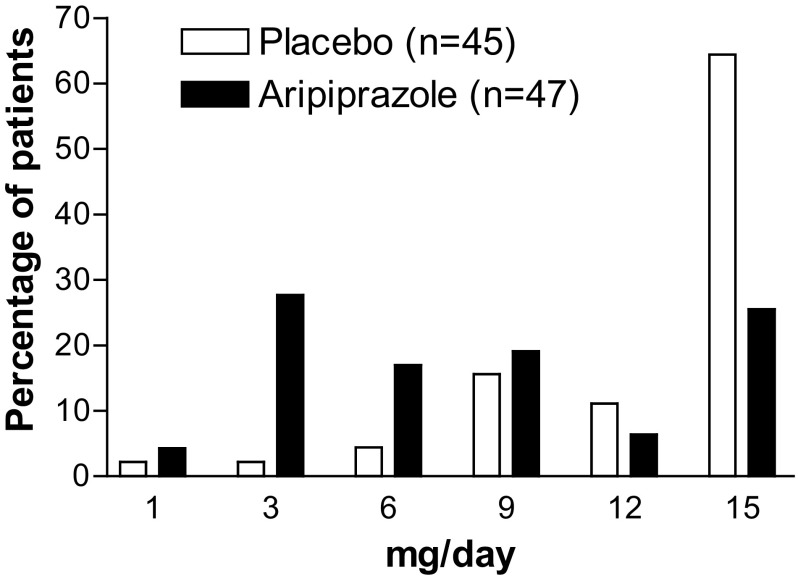
Patient distribution of the last doses of aripiprazole and placebo corresponding to aripiprazole

The mean daily dose of aripiprazole for the whole treatment was 5.7 ± 2.7 mg (±SD). It ranged from 8.1 to 8.4 mg after day 43, at which time the dose was fixed for each patient, with a mean (±SD) daily dose of 8.2 ± 4.9 mg at endpoint. The last dose of aripiprazole (n = 47) was distributed as follows: 1 mg/day, n = 2 (4.3%); 3 mg/day, n = 13 (27.7%); 6 mg/day, n = 8 (17.0%); 9 mg/day, n = 9 (19.1%); 12 mg/day, n = 3 (6.4%); and 15 mg/day, n = 12 (25.5%) (Fig. 2). The mean daily dose of placebo corresponding to aripiprazole was 8.1 ± 2.0 mg (±SD) during the treatment phase. The mean (± SD) daily dose of placebo at endpoint was 12.8 ± 3.6 mg and the prescription pattern of the last dose of placebo corresponding to aripiprazole (n = 45) was distributed as follows: 1 mg/day, n = 1 (2.2%); 3 mg/day, n = 1 (2.2%); 6 mg/day, n = 2 (4.4%); 9 mg/day, n = 7 (15.6%); 12 mg/day, n = 5 (11.1%); and 15 mg/day, n = 29 (64.4%) (Fig. 2).

Hypnotic/sedative/anxiolytic agents [placebo n = 0 (0%); aripiprazole n = 2 (4.3%)] were used concomitantly during the study. Antiparkinson agents were not used by any enrolled patients during the study period.

### Efficacy Outcomes

**Fig. 3:**
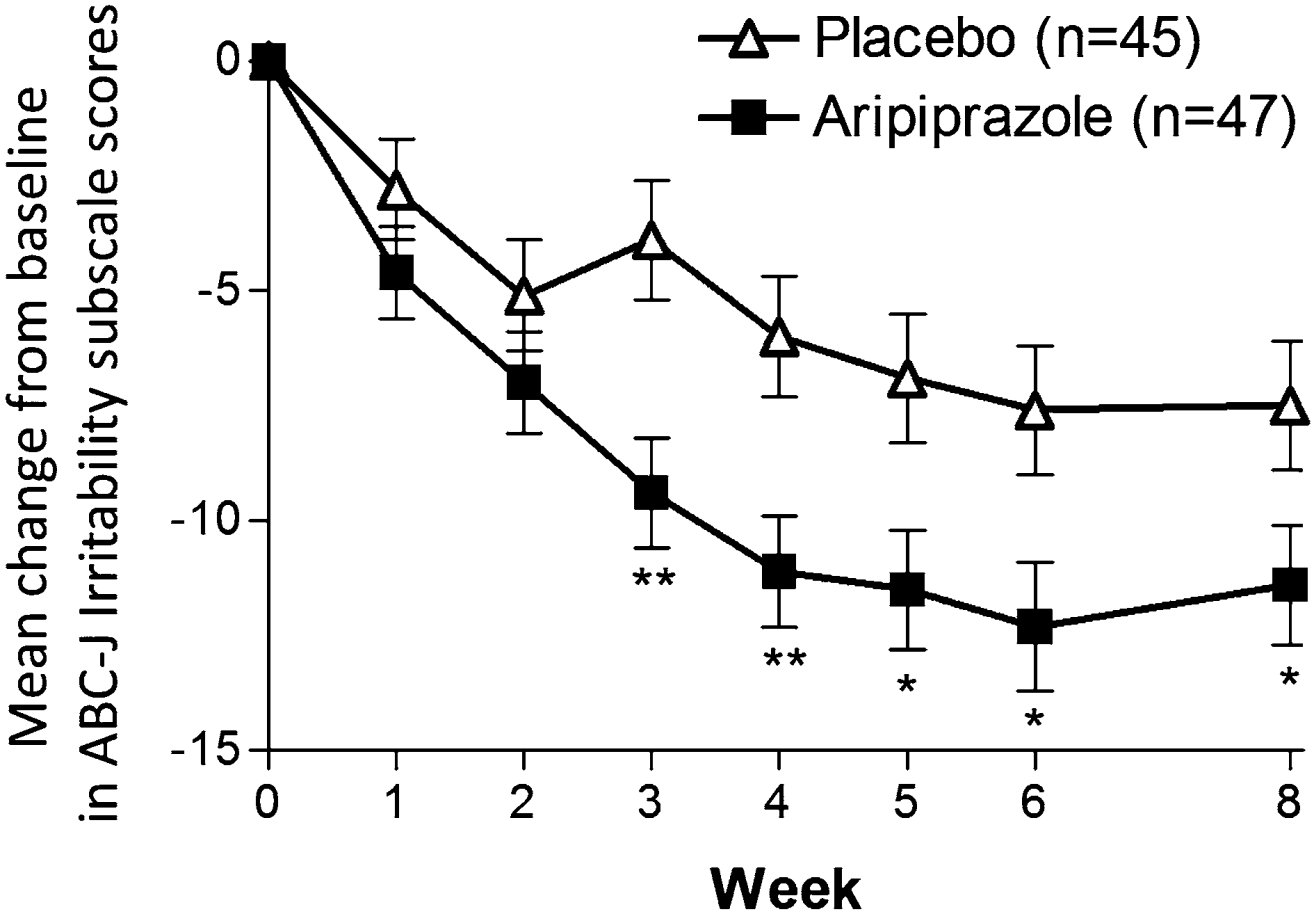
Mean change from baseline in ABC-J Irritability score by week (LOCF; efficacy sample). Data are expressed as least squares mean (standard error). *ABC-J* Aberrant Behavior Checklist Japanese Version, *LOCF* last observation carried forward. *P < 0.05; **P < 0.01 versus placebo

**Fig. 4:**
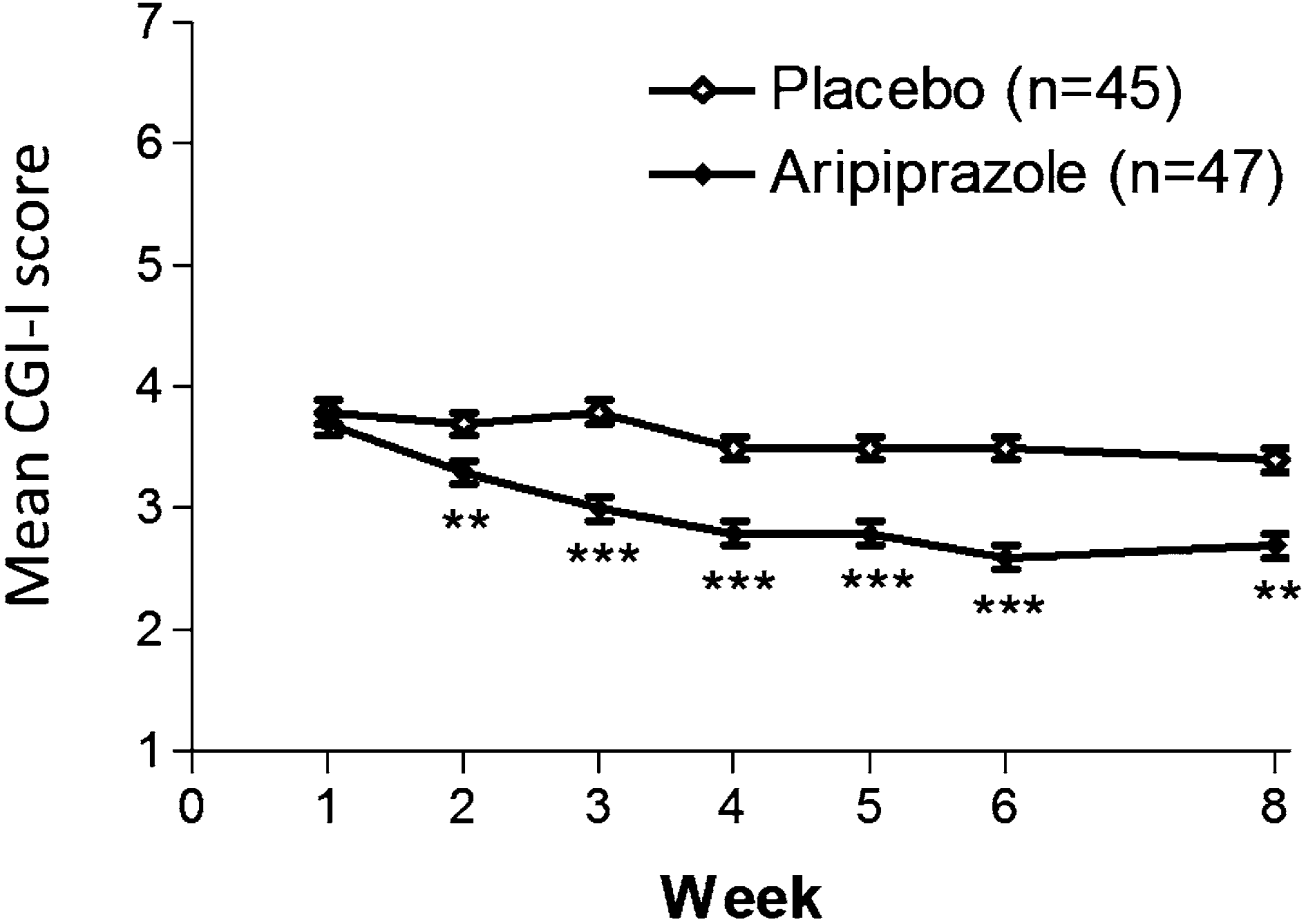
Mean CGI-I score by week (LOCF; efficacy sample). Data are expressed as least squares mean (standard error). *CGI-I* clinical global impressions-improvement score, *LOCF* last observation carried forward. **P < 0.01; ***P < 0.001 versus placebo

**Fig. 5:**
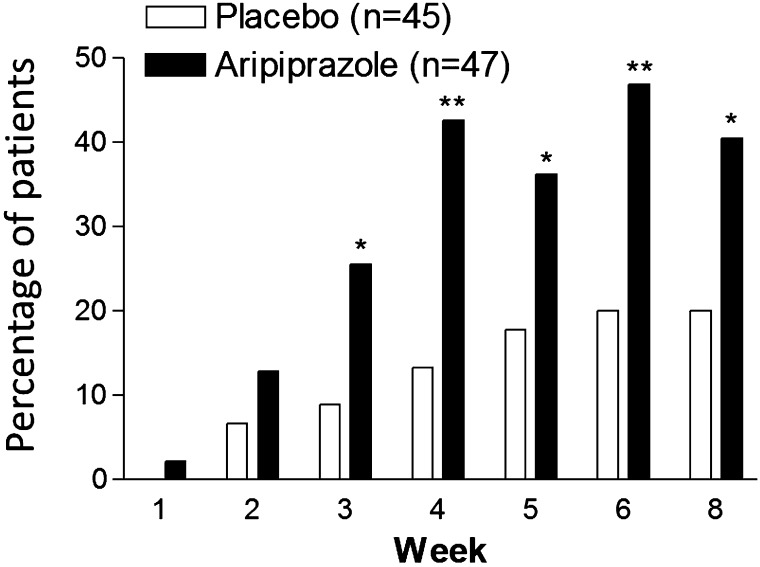
Treatment response rate by week (LOCF; efficacy sample). The treatment response was defined as ≥25% reduction from baseline in the Aberrant Behavior Checklist Japanese Version Irritability subscale score and a Clinical Global Impressions-Improvement score of 1 or 2. *LOCF* last observation carried forward. *P < 0.05; **P < 0.01 versus placebo

Table 2 shows the results for primary and secondary endpoints. At week 8, the least square mean decrease from baseline in the parent/caregiver-rated ABC-J irritability subscale score was significantly greater for patients who received aripiprazole (Table 2; Fig. 3). The TD was −3.94 showing statistical significance (Table 2). Significant TDs in favor of aripiprazole were detected from week 3 through week 8 (Fig. 3). At week 8, aripiprazole group showed a statistically significant improvement in mean clinician-rated CGI-I scores greater than the placebo group [2.7 (0.1) vs 3.4 (0.1); TD: −0.62 (95% CI: −1.02 to − 0.22); p = 0.003]. Significant TDs in favor of aripiprazole were observed from week 2 through week 8 (Fig. 4). Response rates were significantly greater for patients who received aripiprazole from week 3 through week 8 (Table 2; Fig. 5). Patients who received aripiprazole demonstrated significant improvement versus placebo on the mean ABC-J hyperactivity subscale score. Subjects treated with aripiprazole demonstrated statistically significant improvement compared to placebo in the mean CGI-S score from week 2 through week 8 and the CGAS score at weeks 4 and 8. There was no significant difference between aripiprazole group and the placebo group in the mean ABC-J stereotypy, inappropriate speech and lethargy/social withdrawal subscale scores, as well as ABC-J response rate, CY-BOCS (compulsion scale) (Table 2).

**Table 2:** Result of efficacy endpoints (Week 8 LOCF, Efficacy sample)

	Placebo (n = 45)		Aripiprazole (n = 47)		Difference or ratio (95% confidence interval) P value
	Mean baseline	Mean change from baseline	Mean baseline	Mean change from baseline	
ABC-J irritability subscale	26.1 (1.0)	−7.5 (1.4)	26.9 (1.0)	−11.4 (1.3)	−3.94 (−7.77, −0.12)^b^ 0.044
Response rate		9 (20.0)^a^		19 (40.4)^a^	2.02 (1.02, 3.99)^c^ 0.033
ABC-J hyperactivity subscale	26.8 (1.6)	−5.5 (1.5)	29.6 (1.5)	−13.0 (1.4)	−7.55 (−11.53, −3.57)^b^ <0.001
ABC-J stereotypy subscale	7.7 (1.0)	−2.6 (0.6)	8.2 (1.0)	−3.3 (0.6)	−0.67 (−2.42, 1.08)^b^ 0.450
ABC-J inappropriate speech subscale	7.3 (0.6)	−1.5 (0.4)	7.6 (0.5)	−2.2 (0.4)	−0.77 (−1.94, 0.41)^b^ 0.197
ABC-J lethargy/social withdrawal subscale	14.8 (1.4)	−4.7 (1.1)	15.0 (1.4)	−5.2 (1.0)	−0.44 (−3.40, 2.51)^b^ 0.768
ABC-J response rate		12 (26.7)^a^		20 (42.6)^a^	1.60 (0.89, 2.87)^c^ 0.110
CGI-S	5.0 (0.1)	−0.7 (0.2)	4.9 (0.1)	−1.4 (0.1)	−0.62 (−1.03, −0.21)^b^ 0.003
CY-BOCS (compulsion scale only)	5.4 (0.9)	−1.3 (0.5)	6.3 (0.9)	−2.0 (0.5)	−0.69 (−2.03, 0.66)^b^ 0.311
CGAS	42.3 (2.3)	4.5 (1.4)	42.9 (2.2)	9.8 (1.3)	5.25 (1.53, 8.96)^b^ 0.006

Data are expressed as least squares mean (standard error). Response rate is defined as ≥25% reduction from baseline in the ABC-J irritability subscale score and a CGI-I score of 1 or 2. ABC-J response rate is defined as ≥50% reduction from baseline in at least 2 subscales and <10% increase from baseline in the remaining subscales

*CY-BOCS* Children’s yale-brown obsessive–compulsive scale

^a^Number of patients (%)

^b^Difference between aripiprazole and placebo

^c^Ratio between aripiprazole and placebo

### Safety Outcomes

During the study, 33 (73.3%) patients in the placebo group and 39 (83.0%) patients in the aripiprazole group experienced at least one AE. AEs that occurred at an incidence ≥5% in any treatment group are shown in Table 3. All AEs were mild to moderate in severity in both treatment groups. No deaths were reported. Discontinuation due to AEs (exacerbation of the ASD) occurred in 1 (2.2%) patient in the placebo group. A serious AE (heat disorder) occurred in 1 (2.2%) patient in the placebo group. No serious adverse events were reported in the aripiprazole group, in which all patients completed the study.

**Table 3:** Treatment-Emergent Adverse Events that occurred in ≥5% of patients in any group

	Placebo (n = 45)	Aripiprazole (n = 47)
Adverse event	33 (73.3)	39 (83.0)
Somnolence	4 (8.9)	24 (51.1)
Nasopharyngitis	11 (24.4)	10 (21.3)
Decreased appetite	1 (2.2)	6 (12.8)
Nausea	1 (2.2)	3 (6.4)
Vomiting	0 (0.0)	3 (6.4)
Fatigue	0 (0.0)	3 (6.4)
Gastroenteritis	4 (8.9)	1 (2.1)
Bruise	3 (6.7)	1 (2.1)

Data are expressed as number (%)

EPS-related AEs were found in 1 (2.2%) patient in the placebo group and in 3 (6.4%) patients in the aripiprazole group. In the aripiprazole group, EPS-related AEs were observed in 2 (4.3%) patients (salivary hypersecretion) or 1 (2.1%) patient each (akathisia, gait disturbance, bradykinesia, lisp and tremor). One patient in the aripiprazole group experienced salivary hypersecretion, gait disturbance, bradykinesia, lisp and tremor. One (2.2%) patient in the placebo group experienced salivary hypersecretion as EPS. The severity of all observed EPS-related AEs were considered mild in both groups. Mean serum prolactin concentrations at baseline were 16.7 ng/mL for placebo and 14.3 ng/mL for aripiprazole. Aripiprazole was associated with a significant decrease in serum prolactin concentrations compared with placebo from baseline to week 8 (−13.8 vs −2.0 ng/mL; p < 0.001). No patient in the aripiprazole group experienced increase in serum prolactin concentrations. Sedation was reported as an AE in 2.1% (n = 1) of the aripiprazole group and no patients in the placebo group. Increased appetite was reported as an AE in 4.3% (n = 2) of the aripiprazole group and 2.2% (n = 1) in the placebo group. Weight gain was reported as an AE in 2.2% (n = 1) of the placebo group and no patients in the aripiprazole group.

An increase in body weight of ≥7% from baseline was seen in 6.7% of the placebo group and 27.7% of the aripiprazole group. However, the mean change in weight from baseline to week 8 was not significantly different between aripiprazole and placebo (1.24 vs 0.58 kg; p = 0.085). The mean change in BMI from baseline to week 8 was significantly different between aripiprazole and placebo (0.40 versus 0.03 kg/m^2^; p = 0.035). After treatment, the incidence rates of total cholesterol ≥200 mg/dL were: placebo, 9.1% and aripiprazole, 17.0%. No patients experienced fasting blood glucose ≥115 mg/dL, non-fasting blood glucose ≥200 mg/dL, fasting triglyceride ≥200 mg/dL, non-fasting triglyceride ≥500 mg/dL. No marked abnormalities were observed in the other clinical laboratory tests along with vital sign assessments and the 12-lead ECG, except for the decrease in serum prolactin concentrations in the aripiprazole group as described above. There were no adverse events regarding treatment-related suicidal ideation and behavior during the treatment phase.

## Discussion

This 8-week, randomized, multicenter, double-blind, placebo-controlled study demonstrates that flexible-dose aripiprazole can be effective in reducing irritability in Japanese children and adolescents with ASD who manifest a varying range of irritability including tantrums, aggression, and self-injurious behavior. In this study, aripiprazole was significantly more efficacious than placebo at treating irritability, as measured on the caregiver-rated ABC-J irritability subscale from week 3 through week 8. Aripiprazole also produced significant improvements over placebo on the key secondary efficacy measure, the clinician-rated CGI-I score from week 2 through week 8. In addition, there were significantly more responders in the aripiprazole group than in the placebo group. The statistically significant reduction in irritability symptoms (as measured by the ABC-J irritability subscale) is also accompanied by clinically significant results (as measured by the CGI-I). No serious adverse event was reported in the aripiprazole group, in which all patients completed the study. These results showed that aripiprazole was effective, generally safe and well-tolerated in the treatment of irritability associated with ASD in Japanese children and adolescents. Although this study was conducted to clarify new treatment options in specific populations considering influence of genetic/physiologic variation and/or cultural and environmental difference on treatment response of aripiprazole, as a consequence, the results were consistent with those from previous studies done in the US, which showed that aripiprazole is an effective treatment for controlling irritability associated with ASD [8, 9]. With antipsychotic medications, children and adolescents seem to have a higher risk than adults for experiencing adverse events such as EPS, prolactin elevation, sedation, weight gain, and metabolic effects [19]. In our study, the incidence rates of EPS, prolactin elevation, sedation and weight gain in the aripiprazole group were low and the incidence rates of clinically significant changes in triglyceride and blood glucose in the aripiprazole group were low. The efficacy and safety profile of aripiprazole suggested additional benefits of aripiprazole in the pediatric population. Nevertheless, it should be noted that ABC-J does not fully capture the core symptom domains seen in ASD. Aripiprazole also produced greater improvement than placebo in the measure of general functioning at weeks 4 and 8, as measured on the CGAS score. Improvement of psychological, social and school functioning is a meaningful therapeutic goal as the course of treatment of ASD. Therefore, this improvement by aripiprazole may have a substantial impact on the individuals and their families.

In our results, aripiprazole produced greater improvement in the ABC-J irritability subscale than placebo by week 3, when all of the subjects in the aripiprazole group were administered 1–6 mg/day of the medication. Marcus et al. [8] report that aripiprazole produced greater improvement than placebo by the second week of treatment when pediatric patients (aged 6–17 years) received aripiprazole 5 mg/day. Owen et al. [9] reported similar results as early as the first week of treatment, with pediatric patients receiving aripiprazole 2 mg/day. Those studies, conducted in the US [8, 9] suggest an early improvement with low dose aripiprazole and corroborate our results. In previous studies, patients who received aripiprazole and risperidone demonstrated significant improvement versus placebo in ABC stereotypy and inappropriate speech subscale scores [8, 9, 20]. In the previous fixed-dose study of aripiprazole, aripiprazole 15 mg/day demonstrated significant improvements more than the placebo group in ABC inappropriate speech subscale scores and the CY-BOCS compulsion scale score [8]. However, there were no significant differences between aripiprazole lower dose (5 mg/day and 10 mg/day) groups and placebo group [8]. In our study, there was no significant difference between aripiprazole group and placebo group in these measures. The proportion of subjects whose last dose of aripiprazole was 15 mg/day, the highest dose in this study, was only 25.5%. This may explain that the subject proportion receiving the highest dose of aripiprazole was not sufficient to exert a significant improvement in the averages of ABC stereotypy and inappropriate speech subscale scores and the CY-BOCS compulsion scale score, as compared with the placebo group.

Based on our results and previous randomized controlled trials [7], aripiprazole is considered to be well tolerated in children and adolescents in Japan and the US. In our study, somnolence was the most commonly reported AE in the aripiprazole group, and the frequency was higher than that in previous US studies [8, 9]. It is unclear why reported rate of somnolence was higher in Japanese patients than that observed in patients in the US studies, however, this is usually a well-tolerable AE for patients. Aripiprazole treatment was associated with a greater weight gain compared to placebo. The average of body weight gain in the aripiprazole group was greater than the placebo group, 1.24 and 0.58 kg, however it was not statistically significant. This tendency is similar in previous reports [8, 9]. Mean weight gain in aripiprazole group in our study was slightly lower than that in previous fixed-dose aripiprazole study (OC: aripiprazole 5 mg/day 1.5 kg, aripiprazole 10 mg/day 1.4 kg, aripiprazole 15 mg/day 1.6 kg) [8]. The mean change in BMI (kg/m^2^) in aripiprazole group in our study was 0.40 and slightly lower than that in previous fixed-dose aripiprazole study (OC: aripiprazole 5 mg/day 0.5, aripiprazole 10 mg/day 0.5, and aripiprazole 15 mg/day 0.7) [8]. Weight gain was not reported as an AE in the aripiprazole group in our study. It was reported in the previous fixed-dose aripiprazole study (aripiprazole 5 mg/day 7.7%, aripiprazole 10 mg/day 1.7%, and aripiprazole 15 mg/day 3.7%) [8]. In these studies, no subjects discontinued because of weight gain. In previous risperidone study, risperidone therapy was associated with an average weight gain of 2.7 kg [20]. The weight gain in the risperidone group was associated with a mild increase in appetite (49%) or moderate increase in appetite (24%) [20]. The lower incidence rate of increased appetite in aripiprazole group in our study may explain the lower average weight gain. In our study, the frequency of increased appetite was lower than that in previous US studies [8, 9]. However, weight gain and BMI increase were observed with aripiprazole treatment in Japanese children and adolescents ASD patients as well as in US patients [8, 9]; clinicians who treat children and adolescents with aripiprazole should be aware of the potential for weight gain and monitor weight change and provide appropriate advice when necessary [21]. EPS-related AEs are commonly reported for aripiprazole and three (6.4%) patients in the aripiprazole group experienced EPS-related AEs in this trial, with mild severity. The incidence of EPS-related AEs in our study is lower than in previous studies [8, 9]. This could possibly be due to the lower starting dose of 1 mg/day and subsequent dose of 3 mg/day, as compared to previous studies which started at a higher dose of 2 mg/day, followed by 5 mg/day [8, 9].

The decrease in serum prolactin concentrations detected in aripiprazole treated patients in our trial has been previously reported [8, 9], although its clinical consequences are unknown [22]. It is known that serum prolactin level decrease can be induced by the dopamine D_2_ receptor partial agonism, a major property of aripiprazole [5, 22].

The findings of this study are strengthened by the use of both a parent/caregiver-based rating (ABC irritability subscale) and a clinician-based rating (CGI-I scale), both of which demonstrated consistent improvements. Aripiprazole was generally well tolerated, and completion rate was 100%. The incidence of discontinuation from study treatment as a result of AEs in our study is lower than that in the previous flexible-dose study, in which the study duration and the maximum dose of aripiprazole were same as in our study [9]. This could possibly be due to the lower starting and subsequent dose as compared to that of the previous study. These results suggest that this study’s titration strategy provides an appropriate treatment approach for the use of aripiprazole in this population.

Aripiprazole and risperidone are the only the US-FDA approved medications for treating irritability in ASD, however there are few head-to-head data comparing these agents [23]. The choice between these two medications (dopamine D_2_ receptor partial agonist versus antagonist) should be on the basis of clinical equipoise considering the patient’s preference and clinical profile.

To our knowledge, this is the first large, randomised, placebo-controlled trial to be conducted to evaluate the benefits of aripiprazole for the treatment of Japanese ASD patients. Thus, these findings of the study have important implications for the treatment of ASD in this population. However, there are several limitations to this study. The relatively short study duration limits conclusions as to the longer-term efficacy or safety in this population in Japan, and additional evaluation of the effects of aripiprazole on metabolic parameters is warranted. Following this short-term study, a long-term study is ongoing to verify the efficacy and safety of aripiprazole at flexible doses in treating children and adolescents with irritability associated with ASD in Japan. The flexible-dosing paradigm cannot accurately identify a minimally effective dosage or a maximally tolerated dosage, which should be subjects for another study. In the present study, identification of potential marker of aripiprazole treatment response was not included, which should be investigated in a future research.

## Summary

We evaluated the short-term efficacy and safety of aripiprazole in the treatment of irritability in children and adolescents (6–17 years) with ASD in Japan. Ninety-two patients were randomized to receive either placebo (n = 45) or 1–15 mg/day aripiprazole (n = 47) for 8 weeks. The primary outcome measure was change in ABC-J irritability subscale score.

The incidences of adverse events in placebo and aripiprazole groups were 73.3 and 83.0%, respectively. All patients in aripiprazole group completed the study, and no serious adverse events were reported. Three patients in placebo group discontinued because of adverse events (n = 1) and other reasons (n = 2).

Aripiprazole produced a significant improvement in the mean ABC-J irritability subscale score relative to placebo from week 3 through week 8. The most common adverse event in the aripiprazole group was somnolence (51.1%), but this is usually a tolerable AE for patients.

Aripiprazole was effective and generally safe and well-tolerated in the treatment of irritability associated with ASD in Japanese children and adolescents.
